# Sensor Data Security Level Estimation Scheme for Wireless Sensor Networks

**DOI:** 10.3390/s150102104

**Published:** 2015-01-19

**Authors:** Alex Ramos, Raimir Holanda Filho

**Affiliations:** Graduate Program in Applied Computer Science, University of Fortaleza, Av. Washington Soares 1321, Fortaleza 60.811-905, Brazil; E-Mail: raimir@unifor.br

**Keywords:** wireless sensor networks, network security, security level, security metrics, cryptography, key management, intrusion detection systems

## Abstract

Due to their increasing dissemination, wireless sensor networks (WSNs) have become the target of more and more sophisticated attacks, even capable of circumventing both attack detection and prevention mechanisms. This may cause WSN users, who totally trust these security mechanisms, to think that a sensor reading is secure, even when an adversary has corrupted it. For that reason, a scheme capable of estimating the security level (SL) that these mechanisms provide to sensor data is needed, so that users can be aware of the actual security state of this data and can make better decisions on its use. However, existing security estimation schemes proposed for WSNs fully ignore detection mechanisms and analyze solely the security provided by prevention mechanisms. In this context, this work presents the sensor data security estimator (SDSE), a new comprehensive security estimation scheme for WSNs. SDSE is designed for estimating the sensor data security level based on security metrics that analyze both attack prevention and detection mechanisms. In order to validate our proposed scheme, we have carried out extensive simulations that show the high accuracy of SDSE estimates.

## 1. Introduction

In any network, assessing the security level of transmitted information is an essential step for decision making. Especially in sensor networks, the security level of sensed data allows users to distinguish whether the data is actually secure, so that they can properly decide on its use.

In order to estimate the security level of data from a network, it is necessary to evaluate factors that change dynamically, such as different network configurations (e.g., connectivity) and different security mechanism settings (e.g., cryptographic keys shared by the sensors). Therefore, security level quantification can also help security professionals make optimal decisions regarding network configuration adjustments, regarding the security mechanisms used and their respective settings [1].

The basis for estimating data security level in sensor networks is to assess both the vulnerabilities and strengths of the different security mechanisms installed, such as key management schemes (KMSs) and intrusion detection systems (IDSs) [2]. This means that the impact of an attack in a sensor network depends on the effectiveness and vulnerabilities of the security mechanisms deployed. Quantifying the probability of the resistance of security mechanisms against attacks is exactly the goal of security level estimation activity.

A network that has security mechanisms, but that does not have a system to estimate the security level provided by these mechanisms, may lead users to have a false sense of security. This happens because of the simple fact that a network has security mechanisms, but this does not necessarily imply that this network will be totally safe, exactly due to the vulnerabilities of these mechanisms.

Unlike sensor networks, in traditional networks, there is a considerable amount of research (e.g., [1–3]) and availability of standardized techniques for measuring security based on the evaluation of security mechanisms and on the analysis of network vulnerabilities, such as the Common Vulnerability Scoring System (CVSS) standard [4], which is used to quantify the severity of vulnerabilities. However, as sensor networks have vulnerabilities, attacks and security mechanisms different from those of traditional networks (due to the peculiar characteristics of WSNs, such as limited energy, memory and storage), it is not possible that the solutions proposed for traditional networks can be directly applied to WSNs.

Thus, since sensor network security is a relatively new field compared to traditional network security, there are still few security level estimation schemes proposed so far, such as the works in [5–8]. Moreover, none of these schemes validates the security metrics they propose. Furthermore, these proposals do not address the intrusion detection systems, taking into account only the attack prevention mechanisms. Some of them even propose subjective estimation models, by defining weights that must be set by network administrators based solely on their opinion, as the works in [5,7,8]. Another problem is that some of these works focus on calculating only the security level provided by a single security mechanism, as is the case with the works in [5,8], which only estimate the security provided by the mechanisms proposed by the authors themselves. For all of these reasons, these proposals are not well suited for realistic WSN applications. Hence, a security level estimation scheme that tackles these issues is needed.

In this paper, we propose a new security level estimation scheme for WSNs, the sensor data security estimator (SDSE). SDSE has the following main features:

1. Unlike existing works, which use subjective weights, the security metrics defined by SDSE to estimate security level are calculated based on the configuration parameters of both the sensor network and the security mechanisms present in it and on information about the vulnerabilities and attacks of the sensor network;
2. Both prevention and intrusion detection mechanisms are addressed;
3. Security level estimation is based on various types of security mechanisms, rather than being focused only on a specific mechanism. This allows SDSE to be applied to a larger number of sensor networks compared to other proposals.

The rest of the paper is organized as follows: Section 2 describes some important definitions, the representation of SDSE estimates and assumptions. Section 3 presents the operation of the proposed scheme and its estimation model. Section 4 provides a simulation-based evaluation of SDSE. Section 5 describes related work. Finally, Section 6 concludes this paper.

## 2. Definitions, Representation and Assumptions

This section describes the definitions, representation of SDSE estimates and assumptions.

### 2.1. Definitions

SDSE estimates the security level of each sensor data that arrives at the base station (BS) of the WSN. Generally, sensor data is sent to the BS in response to data queries generated by WSN users. Whenever the base station receives sensor data, SDSE delivers that data, along with its estimated security level, to the requesting user.

The security level estimated by SDSE represents the probability of sensor data being secure even in the presence of attacks. This security level results from the combination of a few security metrics. In turn, these security metrics are defined by SDSE for each security mechanism present in the network to indicate the likelihood of these mechanisms resisting attacks (details are in Section 3.2). In other words, a security metric indicates how likely it is that a mechanism will continue to keep network data secure, even when the sensor nodes are being attacked.

More specifically, the data security level estimated by SDSE corresponds to the security level calculated for the sensor nodes that forwarded that data to the BS, including the sensor that generated the data. Thus, to estimate the security level of the given sensor data, SDSE calculates the security metric for each security mechanism in relation to each node of the route traveled by the data and combines these metrics for obtaining the security level of the data (details are in Section 3.3). Therefore, the data security level varies not only according to the current state of the network and the configuration of security mechanisms, but also according to the route traveled by the data.

### 2.2. Representation of SDSE Estimates

According to [9], it is important that metrics are represented by numeric values or percentage, which are more accurate and can better capture the security state of data than qualitative values. Moreover, qualitative high-medium-low values, besides generating the loss of information, introduce subjectivity to metrics. For that reasons, the security estimates computed by SDSE are probability values between zero and one (inclusive), where zero indicates totally insecure data and one indicates totally secure data.

### 2.3. Assumptions

Since SDSE evaluates data security provided by encryption mechanisms, key management schemes and intrusion detection systems (details are in Section 3.2), we assume that the networks in which SDSE will be installed already have these three security mechanisms properly installed and configured. In this case, when SDSE is installed, it will communicate with the underlying operating system of the WSN in order to automatically recognize the security mechanisms installed and will then communicate with these mechanisms whenever it needs to retrieve their configuration parameters required for calculating the security metrics (details are in Section 3.1).

However, in case a sensor network does not have all three security mechanisms, one of the three following approaches can be executed, depending on the choice of the network administrator:

1. SDSE installs the missing security mechanisms in the network. This is possible because SDSE will have these mechanisms previously embedded in itself;
2. SDSE ignores the missing mechanisms and calculates the security level based solely on the mechanisms present in the WSN. In this case, if network data present a high security level, for example, this will indicate that the data are secure in relation to the attacks addressed by the present mechanisms, but it will not be possible to know whether the data are secure regarding the attacks addressed by the missing mechanisms, since these mechanisms could not be evaluated by SDSE;
3. SDSE simply assigns the value of zero to the security level, since the lack of any one of the three main security mechanisms can make the network totally insecure.

The calculations of SDSE estimates are performed in the base station, which does not require any additional energy consumption from the sensor nodes. To make this possible, SDSE requires security mechanisms that have modules running on the base station. Such a type of mechanism is very common in WSNs [10], given that base stations are much more powerful nodes that can perform computations that cannot be run by sensor nodes. In this case, SDSE can communicate with these core modules located at the base station in order to obtain the parameters necessary for calculating SDSE estimates. These modules running on the base station typically manage the operation of the distributed modules that run in each sensor node of the network.

In addition to these parameters obtained in real time from the security mechanisms, in order to properly compute its estimations, SDSE also needs some “static” information about the mechanisms that cannot be obtained during runtime (for example, the security strength of cryptographic keys; see Section 3.2.1). For that reason, we assume that SDSE will keep a database to store that type of “static” information for several existing WSN security mechanisms. As we discuss in Section 3.2, this type of information required by SDSE is publicly available; therefore, such a database can be easily created and distributed along with SDSE.

## 3. Sensor Data Security Estimator

This section describes our proposed SDSE scheme in detail.

### 3.1. Overview of SDSE

SDSE is a system that must be installed in the base station of the target WSN. Once started, SDSE is able to: (1) recognize the security mechanisms installed in the network; and (2) refer to those mechanisms in order to obtain the information necessary to perform security level calculation.

Action (1) is performed through the communication of SDSE with the operating system of the sensor network (e.g., TinyOS [11] and Contiki [12]). Action (2) is performed through communication of SDSE with the modules and communication interfaces provided by the applications (security mechanisms and routing) running on the operating system.

**Figure 1.**
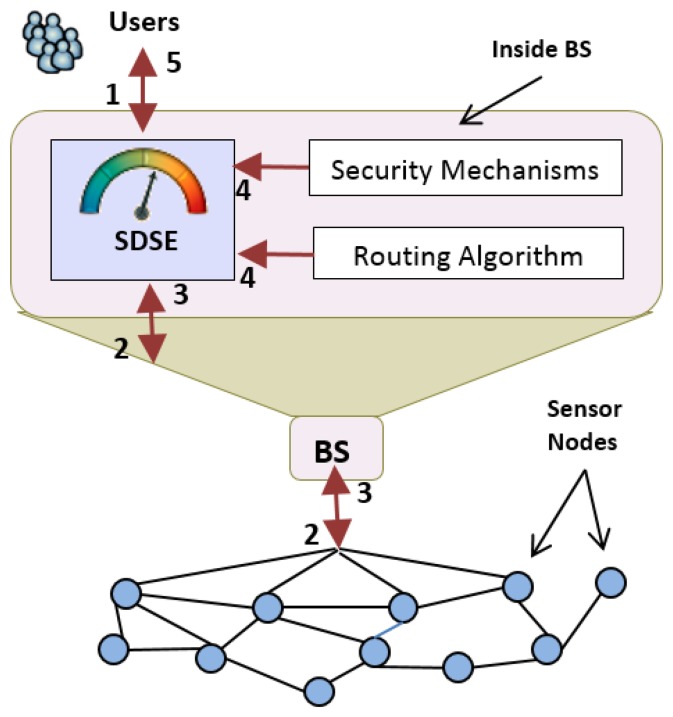
Operation flow of the sensor data security estimator (SDSE).

After being installed, SDSE is ready to calculate the security level of sensed data and report it to the requesting users. In order for SDSE to accomplish this task, the five steps below must be performed (as illustrated in Figure 1):

1. WSN users execute data queries at the base station, in order to obtain certain sensor readings;
2. These user queries are forwarded from the base station to the sensor nodes. This task is generally accomplished by specific query manager modules installed in the base station (omitted from the figure for simplicity), such as TinyDB [13] and Cougar [14], or even data dissemination routing, such as directed diffusion [15];
3. The corresponding sensors send the requested data back to the query manager at the base station;
4. As soon as SDSE receives the sensor readings from the query manager, it communicates with the security mechanisms in order to obtain, at that moment, the parameters needed to calculate the metrics and then communicates with the routing algorithm to discover the route traveled by the received data. With this information, SDSE calculates the security metrics, and from those metrics, it calculates the security level of the received data;
5. Finally, SDSE delivers the sensor data, along with its respective security level, to the requesting user. In possession of the security level received, the user can properly decide on the use of the requested data.

These steps are performed for each query made by any given user. This is necessary, because the data security level constantly changes, since it is calculated based on dynamic parameters, such as network topology, the path traveled by sensor readings and various security parameters, which will be presented in the next section.

### 3.2. Proposed Security Metrics

As stated earlier, SDSE defines a security metric for each considered security mechanism. The three security mechanisms considered by SDSE are: the cryptography algorithm, key management scheme and intrusion detection system. These mechanisms were chosen, because together, they represent the two types of security mechanisms needed to ensure the security of networked systems, which are: prevention mechanisms (represented by the cryptography and the KMS) and detection mechanisms (represented by the IDS). Prevention mechanisms act as a first line of defense, preventing or reducing the potential of attacks. These mechanisms are critical to ensure the main security requirements, such as authentication, confidentiality and integrity. On the other hand, intrusion detection mechanisms act as a second line of defense, identifying and possibly eliminating intruders who evaded prevention mechanisms.

These mechanisms considered by SDSE are the basis for the operation of secondary security mechanisms, such as secure aggregation and secure routing, for example. Therefore, by evaluating the security of the primary mechanisms (cryptography, KMS and IDS), SDSE also addresses the security provided by any other secondary mechanism that include them.

To estimate the security level provided by each of the considered mechanisms, one must initially identify their key vulnerabilities and then define metrics to quantify security, taking into account the impact caused by the exploitation of those vulnerabilities. These details are presented in the following subsections, according to each considered mechanism and the respective security metric defined by SDSE to evaluate it, as listed below:

- The key strength probability metric, for the cryptography algorithm;
- The resilience probability metric, for the key management scheme;
- The legitimacy probability metric, for the intrusion detection system.

#### 3.2.1. Key Strength Probability of the Cryptography Algorithm

**Metric Definition.** The metric *P_f_*(*t*), key strength probability, represents the probability of a cryptographic key (*c*) of a sensor to continue to be secure over time *t*, that is the probability that the key has not been discovered by a brute force attack until time *t.* This probability varies depending on the cryptography algorithm and its security strength for a given key size [16], as described next.

**Features and Vulnerabilities of Cryptography Algorithms**. According to [16], the security “strength” (measured in bits) of an algorithm for a given key size (also measured in bits) is described in terms of the amount of work needed to try all possible keys for a symmetric algorithm with a key size of “X” that has no shortcut attacks. An algorithm that has a Y-bit key, but whose strength is comparable to an X-bit key, of such a symmetric algorithm is said to have a security strength of X bits. Some symmetric-key algorithms have a strength lower than their key size, although most of the algorithms in common use are designed to have a strength equal to their key size. For example, TDEA (triple data encryption algorithm) has a key size of 168 bits, but it provides only 112 bits of security, since an attack of complexity 2^112^ is known. In the case of public key cryptography, an algorithm always has a strength lower than the size of its keys [16].

**Metric Computation.** SDSE calculates *P_F_*(*t*), the probability of key strength of a cryptographic algorithm (for a given key size) at time *t*, as the complement of the probability of that key to be discovered by a brute force attack, *i.e.*, the complement of the success probability of the attack, denoted by *P_A_*(*t*). Therefore:

(1) $$P_{F}(t)=1-P_{A}(t)$$

Thus, by determining *P_A_*(*t*), we can calculate the value of *P_F_*(*t*). For a key that has a strength of *s* bits, the success probability of an attack, *P_A_*(*t*), can be obtained by dividing the number of keys that an adversary was able to try until time *t*, called *k*(*t*), by the total number of possible keys, *i.e.*, 2*^s^*. Therefore:

(2) $$P_{A}(t)=\frac{k(t)}{2^{s}}$$

In order to determine *k*(*t*), we just have to multiply the rate *f* (the number of keys an adversary can test/try per time unit) by time *t*, as shown in the following equation:

(3) k(t)=f⋅t

Substituting Equations (2) and (3) in Equation (1), we can calculate the key strength probability, *P_F_*(*t*), as follows:

(4) $$\begin{array}{cc} P_{F}(t)=1-\frac{k(t)}{2^{s}} & \Leftrightarrow \end{array}$$

(5) $$P_{F}(t)=1-\frac{f\cdot t}{2^{s}}$$

As an algorithm with a security strength of *s* bits will take 2*^s^* · *f*^−1^ time units to have all possible keys tested, after that time, the probability of a key of the network remaining secure is zero. Thus, we can formally define *P_f_*(*t*) as follows:

(6) $$P_{F}(t)=\{\begin{array}{ll} 1-\frac{f\cdot t}{2^{s}} & \text{if}t\leq \frac{2^{s}}{f}\\ 0 & \text{otherwise} \end{array}$$

Hence, for a given test rate *f* and a given security strength *s*, we can compute the key strength probability as a function of time *t*. This means that *P_F_*(*t*) decreases as time goes on, since more keys can be tested at every time unit.

Besides representing the probability of a sensor node key not being discovered by a brute force attack, *P_F_*(*t*) also represents the fraction of network nodes whose keys were not discovered by a brute force attack until time *t*.

**Obtaining Parameters *t, f*** and ***s***. For a given key, the parameter *t* corresponds to the time elapsed from the moment of activation of that key in a given sensor. Each sensor will have its own value of *t*, since nodes have their keys activated at different moments, e.g., when they are added to the network or when their key is changed (rekeying).

Security strength s varies depending on the algorithm and the key size used. Security strength values for symmetric and asymmetric algorithms can be found in periodic reports provided by organizations, such as NIST [16], or projects, like EncryptII [17], among others. In case the security strength of a cryptographic algorithm (for a given key) is unknown, s will correspond to the size of the key used.

Likewise, the value of *f* can be determined according to the algorithm used and will correspond to the higher publicly available key test throughput for that algorithm. For example, the customized machine, Copacobana Rivyera, designed to break the DES (Data Encryption Standard) algorithm, has a throughput *f* = 292 billion keys per second and can find a DES key in less than a single day [18]. The value of *f* is publicly available for each algorithm, but in case this test rate is not publicly known for a given algorithm, its value can be set according to the rates usually achieved when trying to break any algorithm with the same key size using distributed computing (e.g., worms, grid computing or cloud computing), as described in [17].

We highlight that SDSE obtains the parameter *t*, for each node, in execution time, whereas parameters *s* and *f* are gathered from SDSE's database.

**Practical Importance of *P****_F_* (***t***)***.*** The cryptographic strength probability is important to help sensor network administrators choose suitable key sizes that assure the desired level of protection for the network, besides assisting them in deciding on when a sensor key must be renewed. This metric also allows different cryptographic algorithms to be compared with each other. Moreover, as this metric takes into account the time *t* of key activation at each node, it is possible to use this metric in real time in order to inform network users about the security the cryptography provides to each individual sensor node.

**Discussion.** Although in traditional networks, key lengths of 128, 192 and even 256 bits or more can be used, choosing proper key sizes for sensor networks is a critical task, since sensors have limited computational resources and might not always be able to store large keys with high security strengths. Thus, smaller keys have to be employed. For example, TinySec, a security architecture developed for TinyOS, uses the Skipjack algorithm [19] by default, which has a maximum key size of 80 bits. Skipjack is also employed by Minisec [20], another platform developed for TinyOS that provides security for sensor networks. Therefore, since sensor keys are typically small, it is important to have a system like SDSE to monitor, in real time, the status of the security provided by the keys of the sensors nodes.

#### 3.2.2. Resilience Probability of the Key Management Scheme

**Metric Definition.** The metric *P_R_*(*x*), the resilience probability, represents the probability that a secure communication link between any two nodes, A and B, remains secure, even if *x* nodes (not including A and B) have been physically captured by attackers.

**Features and Vulnerabilities of Key Management Schemes.** Key management is a mechanism responsible for the maintenance and distribution of cryptographic keys among nodes in a sensor network. Nodes that share common keys are able to communicate through a secure connection.

As sensor nodes can be deployed in hostile environments, it is possible that an adversary physically captures one or more nodes and gains access to their cryptographic keys. Thus, nodes that share keys with captured nodes may also have their secure connections compromised.

The security provided by key management schemes can be measured on the basis of their resilience toward node capture. The resilience of a key management scheme represents the probability that a secure connection between any two nodes, A and B, is not compromised when x nodes (different from A and B) are captured. The higher the resilience of a key management scheme, the more secure the network.

**Metric Computation.** SDSE computes *P_R_*(*x*), the resilience probability of a communication link between two nodes when *x* other nodes are captured, by the following equation:

(7) $$P_{R}(x)=1-P_{C}(x)$$

where *P_C_*(*x*) represents the probability that a link is compromised when *x* nodes are captured. *P_C_*(*x*) is defined by the following conditional probability:

(8) $$P_{C}(x)=P\left\{L_{c}|C_{x}\right\}$$

where *L_c_* is the event in which a link is compromised and *C_x_* is the event in which *x* nodes are captured.

For each key management scheme, a specific equation to calculate *P_C_*(*x*) exists, since each scheme operates differently from the others and is based on different configuration parameters. These equations are usually defined and published by the authors of each management scheme, since they are usually quite complex. In [21], Simplício Jr. *et al.* present various key management schemes and their respective equations to calculate *P_C_*(*x*) when a single node is captured (*i.e.*, *x* = 1).

In the following subsection, we give an example of a key management scheme and show how to calculate its respective probability *P_C_*(*x*), which can then be used by SDSE to compute the resilience probability *P_R_*(*x*) for that scheme, via Equation (7).

**Example of *P****_C_*(***x***) **Computation.** In the q-composite random key pre-distribution scheme (proposed by Chan *et al.* [22]), a scheme on which several other schemes are based, initially, a set *K* of keys is randomly chosen from the total possible key space. This key pool is generated offline and has size of |*K*|*.*

For each node in the sensor network, *k* keys are randomly picked from the key pool *K* and stored in the node's memory. This subset of *k* keys is called the node's key ring. The size of the key pool, |*K*|, is chosen, such that there is a probability *p* that two random subsets of size *k* in *K* share at least one key.

After network deployment, every node shares information with its neighbors in order to find out which keys they have in common. If a node has at least *q* keys in common with a neighbor, it is said that they share a secure communication link, *i.e.*, two nodes have a secure communication link between them when they share *i* keys, where *q* ≤ *i* ≤ *k.* In this case, the secure communication key *c* (the key that is also vulnerable to brute force attacks, defined in Section 3.2.1) is generated by the hash of all keys in common, that is, *c* = *hash*(*k*_1_ ‖ *k*_2_‖… ‖*k_i_*)*.* Neighboring nodes that share less than *q* keys cannot establish a secure communication link.

Depending on the desired probability of connection *p* and the number of keys *k* stored by each node, the size of the key pool |*K*| can be determined. In other words, for nodes storing *k* keys on their key ring, determine what should be the appropriate size of *K*, such that the probability of any two nodes sharing *q* keys in common is ≥ *p.*

Chan *etal.* [22] show that the key pool size |*K*| can be computed as follows. Let *p*(*i*) be the probability that any two nodes in the network have exactly ***i*** keys in common. Any given node has $\left(\begin{array}{c} \left|K\right|\\ k \end{array}\right)$ distinct ways of choosing *k* keys from the key pool of size |*K*|*.* Therefore, ${\left(\begin{array}{c} \left|K\right|\\ k \end{array}\right)}^{2}$ is the total number of ways of any two nodes choosing *k* keys each. If we suppose that these two nodes have *i* common keys, then $\left(\begin{array}{c} \left|K\right|\\ i \end{array}\right)$ is the number of ways for both nodes to choose the *i* keys in common. Once the *i* common keys have been selected, 2(*k*−*i*) distinct keys in the two key rings are yet to be selected from the remaining key pool of size |*K*|−*i*. This can be done in $\left(\begin{array}{c} \left|K\right|-i\\ 2(k-i) \end{array}\right)$ different ways. Next, the 2(*k*−*i*) distinct keys must be equally divided between the two nodes. This division can be done in $\left(\begin{array}{c} 2\left(k-i\right)\\ \left(k-i\right) \end{array}\right)$ different ways. Hence, the total number of ways one can choose two key rings with ***i*** keys in common is the product of the three terms previously mentioned, that is $\left(\begin{array}{c} \left|K\right|\\ i \end{array}\right)\left(\begin{array}{c} \left|K\right|-i\\ 2\left(k-i\right) \end{array}\right)\left(\begin{array}{c} 2\left(k-i\right)\\ \left(k-i\right) \end{array}\right)$ . Hence, we have:

(9) $$p(i)=\frac{\left(\begin{array}{c} \left|K\right|\\ i \end{array}\right)\left(\begin{array}{c} \left|K\right|-i\\ 2\left(k-i\right) \end{array}\right)\left(\begin{array}{c} 2\left(k-i\right)\\ \left(k-i\right) \end{array}\right)}{{\left(\begin{array}{c} \left|K\right|\\ k \end{array}\right)}^{2}}$$

Let *p_connect_* be the probability that any two nodes have sufficient keys in common to establish a secure connection. Hence, we have *p_connect_* = **1**− (the probability that the two nodes have insufficient keys in common to establish a secure connection). Thus:

(10) $$P_{connect}=1-\left(p(0)+p(1)+\ldots +p(q-1)\right)$$

Hence, for a given key ring size *k*, minimum key overlap *q* and minimum connection probability *p*, the largest |*K*| should be chosen, such that *p_connect_* ≥ *p*.

Chan *et al.* show that the value of parameters *k*, *q* and *p* must be chosen according to the network size and the average neighborhood size of the nodes. Once these values are set, for a given network, the value of |*K*| can then be computed, and the network can, therefore, be deployed. In such a network, given the fact that nodes share common keys, the capture of *x* nodes can compromise the secure communication links between nodes, which are not included in the *x* captured nodes.

Our aim is to compute *P_C_*(*x*) (the probability that any given link is compromised when *x* nodes are captured) for the q-composite scheme. Chan *et al.* compute *P_C_*(*x*) as follows. Since each node has *k* keys, the probability that a given key has not been compromised is ${\left(1-\frac{k}{\left|K\right|}\right)}^{x}$ . The expected fraction of total keys compromised is thus $1-{\left(1-\frac{k}{\left|K\right|}\right)}^{x}$ . For any secure communication link between two nodes, if its link key was the hash of *i* common keys, then the probability of that link being compromised is ${\left(1-{\left(1-\frac{k}{\left|K\right|}\right)}^{x}\right)}^{i}$ . The probability of establishing a secure link is *p* = *p*(*q*) + *p*(*q* + 1) + … + *p*(*k*)*.*

Therefore, for q-composite:

(11) $$P_{C}(x)=\sum_{i=q}^{k}{\left(1-{\left(1-\frac{k}{\left|K\right|}\right)}^{x}\right)}^{i}\frac{p(i)}{p}$$

This equation also represents the fraction of secure communications independent of the captured nodes that an adversary can compromise based on the information retrieved from the *x* captured nodes.

With the equation above, SDSE can calculate the resiliency probability *P_R_*(*x*) for the q-composite scheme applying Equation (7).

**Obtaining Parameters *k*,** ***q*, *p*, *K*** and ***x.*** As can be seen in [22], the values of *k*, *q*, *p* and *K* are based on the network size and the average neighborhood of the nodes. They can be set by the network administrator directly into q-composite, which, in turn, can pass them to SDSE. This also applies to other key management mechanisms, which, like the q-composite scheme, are able to provide their parameters to SDSE.

The value of *x* can be obtained by SDSE through any node capture identification mechanism embedded in the key management scheme of the network, such as the model based in sequential analysis proposed in [23], which identifies node captures that usually cannot be identified by the intrusion detection system of the network.

**Practical Importance of** ***P_R_***(***x***)**.** The resilience probability is important to help WSN administrators choose suitable key management schemes for their network and properly set up their parameters in order to provide the desired security level. Moreover, as this metric takes into account the amount of attacks in the network at any given moment, it can be used in real time to inform network users about the status of the security provided by the key management scheme to the communication links of each sensor node.

#### 3.2.3. Legitimacy Probability of the Intrusion Detection System

**Metric Definition.** SDSE defines legitimacy probability (*P_L_*) as the chance of a node being legitimate (that is, not malicious), given the result of the evaluation of the security status of that node performed by the IDS.

IDS's evaluation of a given node can have two possible results: (1) the node is identified as malicious and an alarm is raised (*A*+); or (2) the node is identified as legitimate and no alarm is raised (*A*−)*.* In turn, a node can have two possible states: (I) malicious (*M*+); or (II) legitimate (*M*−). Thus, depending on the outcome of the IDS, *P_L_* can be computed by one of the two following ways:

- ***P****_L_*(***alarm***) = ***P***(***M* −** |***A***+)***:*** the probability that a node is legitimate (*M*−), given that the IDS has raised an alarm (*A*+) for that node or;
- ***P****_L_*(¬***alarm***) = ***P***(***M* −** | ***A*−**)**:** the probability that a node is legitimate (*M*−), given that the IDS has not raised an alarm (*A*−) for that node.

**Features and Vulnerabilities of Intrusion Detection Systems.** Intrusion detection systems analyze network traffic in order to identify malicious activity. Whenever an attack is identified, the IDS raises an alarm to inform the base station. In fact, given the distributed nature of sensor networks, it is interesting that the intrusion detection process is performed by the network nodes themselves, in a distributed and cooperative manner. Such an approach allows the IDS (as a whole) to deal with malicious nodes that try to either hide from detection or raise false intrusion alerts.

More specifically, in a distributed, cooperative detection approach, each node monitors its neighborhood, looking for suspicious behavior. Once a suspicious activity is detected, a collaborative process in which neighboring nodes exchange information about the suspected node is started. In this collaborative process, each neighbor node must indicate whether they consider the suspected node as malicious or not. This indication is normally performed via the generation of individual alarms, *i.e.*, in order to indicate that a suspected node is malicious, a neighbor will raise an alarm (*a*+) for that node. On the other hand, in order to indicate that a suspected node is not malicious, a neighbor will not raise any alarm (*a*−) for that node.

At the end of the collaborative process, a suspected node is classified by the IDS as truly malicious when at least m (consensus parameter) of its *N* neighbors has raised an alarm (*a*+) for it. If this is the case, the IDS generates a global alarm (*A*+) for the suspected node and sends it to the base station, so that the appropriate counter measures can be taken.

However, intrusion detection systems are subject to errors in the detection process and can, therefore, either incorrectly accuse legitimate nodes of being malicious or mistakenly classify malicious nodes as legitimate. Given the two possible states of a node, malicious (*M*+) or legitimate (*M*−), the two possible outcomes of each individual neighbor, alarm (*a*+) or no alarm (*a*−), can be classified into one of the four following cases:

1. True positive (TP): alarm properly generated for a malicious node;
2. False positive (FP): alarm wrongly generated for a legitimate node;
3. False negative (FN): alarm wrongly not generated for a malicious node;
4. True negative (TN): alarm properly not generated for a legitimate node.

As the evaluation is initially performed individually by each neighbor, one can run some experiments in order to compute the amount of hits and misses performed by each neighbor. This can be done in a controlled environment in which the attacks are known beforehand. In such experiments, several attacks are performed on the sensor network, and the outcomes of the evaluations performed by each individual neighbor are then classified into one of the four possible cases mentioned (TP, FP, FN, TN). From these values, we can compute the miss and hit rates of each neighbor. There are four rates to assess the effectiveness of the evaluations performed by an individual neighbor, one for each of the four possible cases mentioned, namely:

- True positive rate (***P****_tp_*): This represents the fraction of malicious nodes for which this neighbor has generated an alarm. This rate also represents the probability that this neighbor generates an alarm (*a*+) for a malicious node (*M*+), *i.e.*, probability $P\left(a+|M+|\right)=\frac{TP}{TP+FN}$ ;
- False positive rate (***P****_fp_*): This represents the fraction of legitimate nodes for which this neighbor has generated an alarm. This rate also represents the probability that this neighbor generates an alarm (*a*+) for a legitimate node (*M*−), *i.e.*, probability $P\left(a+|M-\right)=\frac{FP}{TN+FP}$ ;
- False negative rate (***P****_fn_*): This represents the fraction of malicious nodes for which this neighbor has not generated an alarm. This rate also represents the probability that this neighbor does not generate an alarm (*a*−) for a malicious node (*M*+), *i.e.*, probability $P\left(a-|M+\right)=\frac{FN}{TP+FN}$;
- True negative rate (*P_tn_*): This represents the fraction of legitimate nodes for which this neighbor has not generated an alarm. This rate also represents the probability that this neighbor does not generate an alarm (*a*−) for a legitimate node (*M*−), *i.e.*, probability $P\left(a-|M-\right)=\frac{TN}{TN+FP}$.

Notice that the true positive rate is the complement of the false negative rate $P_{tp}=P_{fn}^{C}$ . Likewise, the false positive rate is the complement of the true negative rate $\left(P_{fp}=P_{tn}^{C}\right)$ , that is:

(12) $$\begin{array}{ccc} P_{tp}=1-P_{fn} & \text{e} & P_{fp}=1-P_{tn} \end{array}$$

Since the result of a global evaluation performed by the IDS is given by the combination of the results of individual evaluations performed by the neighbors of a suspected node, the hit and miss rates of a global evaluation depend on the amount of neighbors (*N*) and the consensus parameter (*m*)*.* Thus, in a real WSN, one can estimate the hit and miss rates of global evaluations from the hit and miss rates of the individual neighbors, which were obtained by means of experiments in a controlled environment.

Therefore, considering *i* as the number of neighbors that have generated an alarm for a suspected node, in order for a global alarm (*A*+) to be generated for a given node, it is necessary that at least *m* neighbors have generated an alarm for this node, *i.e.*, *m* ≤ *i* ≤ *N.* In this case, the *N* neighbors can be combined into generators and non-generators of an alarm in $\left(\begin{array}{c} N\\ i \end{array}\right)$ different ways. Hence, if the suspected node is actually malicious (*M*+), then the *i* neighbors that have generated an alarm have produced a true positive (TP) outcome, and the *N* −*i* neighbors that have not generated an alarm have produced a false negative (FN) outcome. Thus, assuming that each individual neighbor has the same hit and miss rates, we can compute the global true positive rate (*P_TP_*) (*i.e.*, *P*(*A*+ |*M*+), the probability that a global alarm is generated for a malicious node), from both the individual true positive rate (*P_tp_*) and the individual false negative rate (*P_fn_* = 1−*P_tp_*), by using the following equation based on the binomial distribution:

(13) $$P_{TP}=P\left(A+|M+\right)=\sum_{i=m}^{N}\left(\begin{array}{c} N\\ i \end{array}\right){\left(P_{tp}\right)}^{i}{\left(1-P_{tp}\right)}^{N-i}$$

Likewise, for the case that a global alarm (*A*+) is generated for a suspected node that is actually a legitimate node (*M*−), we compute the global false positive rate (*P_FP_*) (*i.e.*, *P*(*A*+|*M*−), the probability that a global alarm is generated for a legitimate node), from both the individual false positive rate (*P_fp_*) and the individual true negative rate (*P_tn_* = 1−*P_fp_*), by using the following equation based on the binomial distribution:

(14) $$P_{FP}=P\left(A+|M-\right)=\sum_{i=m}^{N}\left(\begin{array}{c} N\\ i \end{array}\right){\left(P_{fp}\right)}^{i}{\left(1-P_{fp}\right)}^{N-i}$$

On the other hand, if we take *j* as the number of neighbors that do not generate an alarm for a suspicious node, for the case that a global alarm is not generated for a node (*A*−), it is necessary that at least *N* − *m* + 1 neighbors have not generated an alarm for that node, *i.e.*, *N* − *m* + 1 ≤ *j ≤ N.* Thus, if the node is actually legitimate (*M*−), then the *N* − *j* + 1 neighbors that have not generated an alarm have produced a true negative (TN) outcome, while the *N* − (*N* − j + 1) = *j* − 1 neighbors, which have generated an alarm, have produced a false positive (FP) outcome. Thus, it is possible to obtain the global true negative rate (*P_tn_*) (*i.e.*, *P*(*A* − |*M*−), the probability that a global alarm is not generated for a legitimate node), from both the individual true negative rate (*P_TN_*) and the individual false positive rate (*P_fp_* = 1 − *P_tn_*), by using the following equation based on the binomial distribution:

(15) $$P_{TN}=P\left(A-|M-\right)=\sum_{j=N-m+1}^{N}\left(\begin{array}{c} N\\ j \end{array}\right){\left(P_{tn}\right)}^{j}{\left(1-P_{tn}\right)}^{N-j}$$

Likewise, for the case that a global alarm (*A*−) is not generated for a suspected node that is actually a malicious node (*M*+), we can compute the global false negative rate (*P_FN_*) (*i.e.*, *P*(*A* − |*M*+), the probability that a global alarm is not generated for a malicious node), from both the individual false negative rate (*P_fn_*) and the individual true positive rate (*P_tp_* = 1 − *P_fn_*), by using the following equation based on the binomial distribution:

(16) $$P_{FN}=P\left(A-|M+\right)=\sum_{j=N-m+1}^{N}\left(\begin{array}{c} N\\ j \end{array}\right){\left(P_{fn}\right)}^{j}{\left(1-P_{fn}\right)}^{N-j}$$

From this information, we can finally proceed to the computation of the legitimacy probability (*P_L_*)*.*

**Metric Computation.** As we have seen, due to the miss rates of individual nodes, the global decision of an IDS may be incorrect. Hence, in order to evaluate the security status of a node, SDSE defines the legitimacy probability (*P_L_*), which represents the chance of a node being legitimate, given the global decision of the IDS. Depending on this decision, for a given node, *P_L_* can be given by either *P_L_*(*alarm*) = *P*(*M* − |*A*+) or by *P_L_*(¬*alarm*) = *P*(*M* − |*A*−), as explained earlier in the definition section of this metric.

These two probabilities can be derived by the application of conditional probability, more precisely Bayes' theorem. Beginning with *P_L_*(*alarm*) = *P*(*M* − |*A*+), we have:

(17) $$P\left(M-|A+\right)=\frac{P\left(A+|M-\right)P\left(M-\right)}{P\left(A+\right)}\Leftrightarrow$$

(18) $$P\left(M-|A+\right)=\frac{P\left(A+|M-\right)P\left(M-\right)}{P\left(A+|M-\right)P\left(M-\right)+P\left(A+|M+\right)P\left(M+\right)}$$

Notice that *P*(*A* + |*M*−) corresponds to the global false positive rate (*P_FP_*, Equation (14)), and *P*(*A* + |M+) corresponds to the global true positive rate (*P_TP_*, Equation (13)). On the other hand, *P*(*M*+), also known as the base rate [24], corresponds to the fraction of malicious nodes in the network, *i.e.*, the probability that a randomly selected node from the network is malicious (*P_M_*)*.* Lastly, note that *P*(*M*−) = 1 − *P*(*M*+), *i.e.*, *P*(*M*−) = 1 − *P_M_*. Therefore, the legitimacy probability of a node for which a global alarm has been generated, *P_L_*(*alarm*), can be given by the following equation:

(19) $$P_{L}\left(alarm\right)=P\left(M-|A+\right)=\frac{P_{FP}\left(1-P_{M}\right)}{P_{FP}\left(1-P_{M}\right)+P_{TP}P_{M}}$$

Substituting the values of *P_TP_* and *P_FP_* by their respective Equations (13) and (14), *P_L_*(*alarm*) can be directly derived from the respective hit and miss rates of individual neighbors of a suspected node (*P_tp_* and *P_fp_*)*.*

Similarly, we have the following equation for the metric *P_L_*(¬*alarm*) = *P*(*M* − |*A*−) by applying Bayes' theorem:

(20) $$P_{L}\left(\neg alarm\right)=P\left(M-|A-\right)=\frac{P_{TN}\left(1-P_{M}\right)}{P_{TN}\left(1-P_{M}\right)+P_{FN}P_{M}}$$

Substituting the values of *P_TN_* and *P_FN_* by their respective Equations (15) and (16), the metric *P_L_*(¬*alarm*) can be directly derived from the respective hit and miss rates of individual neighbors of a suspicious node (*P_tn_* and *P_fn_*).

**Obtaining Parameters m, *N***, ***P****_tp_*, ***P****_fp_*, ***P****_tn_****, P****_fn_* and ***P****_M_***.** The consensus parameter *m* is set up by the network administrator directly in the IDS, which, in turn, can pass it to SDSE. The number of neighbors of a node (*N*) can be obtained by SDSE from the routing algorithm of the WSN. The hit rates (*P_tp_* and *P_tn_*) and miss rates (*P_fp_* and *P_fn_*) of individual neighbors can be obtained by means of experiments in a controlled environment in which attacks are known in advance. In fact, these individual rates are usually published by the authors of each intrusion detection system proposed for sensor networks, but they can also be computed by WSN security administrators.

The probability that a given node of the network is malicious, *P_M_*, can be easily obtained through offline traffic analysis constantly performed by the IDS itself at a central point, such as the approaches proposed in IDSs [25,26].

We highlight that SDSE obtains parameters *m*, *N* and *P_M_* in execution time, whereas parameters *P_tp_*, *P_fp_*, *P_tn_* and *P_fn_* are gathered by SDSE from its database.

**Practical Importance of *P****_L_***.** This metric can be used offline to help security professionals choose the appropriate value for m, according to the amount of neighbors (*N*) of the nodes of the network, in order to ensure the desired security level for the network. Furthermore, *P_L_* can be used to evaluate the impact of parameters *P_tp_*, *P_fn_*, *P_tn_*, *P_fp_* and *P_M_* on the security of the sensors. Moreover, this metric can also be used to compare different intrusion detection systems. When used in real time, this metric can inform users about the security of sensors, which varies with the number of neighbors of a node (*N*), the consensus parameter *m* and the hit and miss rates inherent to the IDS of the network.

**Discussion.** The value of the network base rate (*P_M_*) can greatly vary over time, given that detection mechanisms are able to perform counter measures, such as permanently isolating the malicious nodes from the network through cryptographic keys revocation [21,27,28] or simply healing (recovering) the malicious nodes. There are several situations in which malicious nodes can be recovered. For example, nodes that behave maliciously due to software failures or software modifications made by an adversary can be turned into legitimate again by application of node reprogramming [29,30]. Reprogramming can be performed remotely by a network administrator at the base station. Recovery methods in both hardware and software for malicious or faulty sensor nodes can be seen in [31].

**Restrictions.** SDSE is designed to work only with hybrid IDSs, such as the ones proposed in [26,32,33], for example. This is required, because only this type of IDS is capable of providing the network base rate (*P_M_*) to SDSE. That is, this type of IDS, in addition to performing distributed, collaborative detection in real-time, can also perform centralized, offline detection, which is responsible for providing *P_M_*. In centralized detection, a great amount of traffic information is sent to the base station, a central point on the network that, differently from sensor nodes, has no resource constraints and can then perform more sophisticated and accurate traffic analyses than those performed by collaborative real-time detection [10]. Although this detailed analysis performed in the base station can be quite time-consuming, it allows not only detecting attacks that have already occurred, but also predicting the network base rate at any given moment based on the historical traffic information. This allows SDSE to obtain, from the IDS, the expected current network base rate in real time (*i.e.*, at the moment it calculates the legitimacy probability *P_L_* for a given node).

Actually, we see the fact that SDSE can only work with hybrid IDSs as an advantage, because, as discussed in [34], hybrids IDSs are the future of intrusion detection in sensor networks, since they have the strengths of both centralized and distributed IDSs.

### 3.3. Security Level Estimation

The main goal of SDSE is to provide the SL of sensor data to WSN users. In order to achieve this goal, SDSE estimates the security of the route by which the data have traveled until reaching the base station. The security of a given route is measured on the basis of the security of the sensors that comprise it. SDSE uses the metrics defined in Section 3.2 to compute the security of the nodes of the route. As stated previously, the security level measured by SDSE is a value in the interval [0, 1], calculated for each sensor datum in order to indicate how secure that datum is.

Typically, sensor data refer to readings performed by sensor nodes sent to the BS in response to a query made by a user. Whenever a query response arrives at the base station, SDSE obtains from the routing protocol information about the route traveled by the response. Furthermore, SDSE obtains from the security mechanisms the parameters necessary to compute the security metrics for each sensor of the discovered route. After computing the security metrics, SDSE combines them in order to obtain the SL.

Formally, for a route *R* = {*n_i_* | *i* = 1,2,…, *q*}, SDSE initially calculates for each node *n_i_* belonging to *R* its security degree (*g_i_*)*.* The value of *g_i_* is calculated as the product of the security metrics computed for node *n_i_*, defined in Equations (6), (7) and (19) (if the node has been accused of being malicious by the IDS or Equations (6), (7) and (20) otherwise). The value of *g_i_* is given by the multiplication of the metrics, because each metric corresponds to an independent event. This is because each security mechanism has different vulnerabilities and deals with different types of attack. The value of *g_i_* is given by the following equation:

(21) $$g_{i}=P_{F}^{i}\times P_{R}^{i}\times P_{L}^{i}$$

Hence, we obtain the set *G* = {*g_i_* | *i* = 1,2,…, *q*}, which contains the security degrees of all nodes of route *R.* As the node having the lowest security degree of the route is the weakest link [35] and is, therefore, more likely to compromise the data, SL is given by the minimum value of *G*, as can be seen in the following equation:

(22) $$SL=\min\left(g_{1},g_{2},\ldots ,g_{q}\right)$$

Lastly, SDSE assigns the computed SL to its respective query response and then deliver both of them to the requesting user.

## 4. Simulations and Results Analysis

In this section, we evaluate the behavior of SDSE's proposed equations and also validate them through extensive simulations.

### 4.1. Methodology

The validation is carried out by measuring the accuracy of SDSE security estimates regarding the actual security status of the simulated networks. More specifically, to measure how close an estimated value (*V_E_*) is from an actual value, measured in a simulation, in this case, (*V_R_*), we use the following equation:

(23) $$\text{Accuracy}=100\times \left(1-\frac{\left|V_{R}-V_{E}\right|}{V_{R}}\right)$$

Since in each of its equations, SDSE uses several configuration parameters of the network security mechanisms, various scenarios have been simulated, so that we could validate SDSE estimates under different circumstances.

Each scenario has been simulated 100 times, and the average result of the obtained accuracies has been considered for analysis. Since in each scenario the results obtained in the 100 simulation runs have presented approximately the same pattern, we conclude that 100 simulation runs can give us reliable results.

### 4.2. Simulation Environment

We have performed the simulations using Castalia [36], a discrete event simulator suitable for sensor networks, based on OMNeT++ [37], a platform for building event-driven simulators. Castalia is a powerful tool able to simulate realistic wireless channel and radio models, with a realistic node behavior.

We have deployed five differently sized WSNs consisting of 200, 400, 600, 800 and 1000 nodes. In each of these networks, there is only one base station, and the nodes are static and are uniformly distributed in a square region. The node density was set to 8 sensors per 100 m^2^, while increasing the region size to fit 200 to 1000 nodes. Each sensor was set up with a radio range of 50 m, assuming bidirectional links. The radio used in the simulations was CC2420, which is used in several real sensors, such as MICAz and TelosB [38].

The MAC layer protocol used is T-MAC [39], a contention-based medium access control protocol suitable for WSNs. In the network layer, we have used the multipath ring routing protocol (provided by Castalia), a simple implementation of the synopsis diffusion protocol [40]. Each network is configured so that every 1 s, for 20 min, each node sends a message to the base station containing four readings (temperature, humidity, pressure and light).

Finally, we highlight that the settings described above have been used in all simulation runs, unless otherwise specified.

### 4.3. Key Strength Probability (*P_F_*)

In this section, we first present the behavior of the metric *P_F_* according to Equation (6). Then, we present the accuracy achieved by *P_F_* in the simulations.

#### 4.3.1. Behavior Analysis of *P_F_*

To show the behavior of *P_F_*(*t*), the key strength probability as a function of t (the time interval elapsed since the activation of the key of a node), we use the RC5 cryptography algorithm [41], because, besides being a widely-used algorithm, it is used in TinySec [42], a security architecture for WSNs implemented in TinyOS [11].

RC5 has a variable key size (0 to 2040 bits). The Distributed Computing Technologies, Inc. (a.k.a. distributed.net) [43] runs several brute force attack projects that aim to break RC5 keys using distributed computing. Some of these projects manage to achieve test rates (*f*) of more than 394 billion keys per second [43].

**Figure 2.**
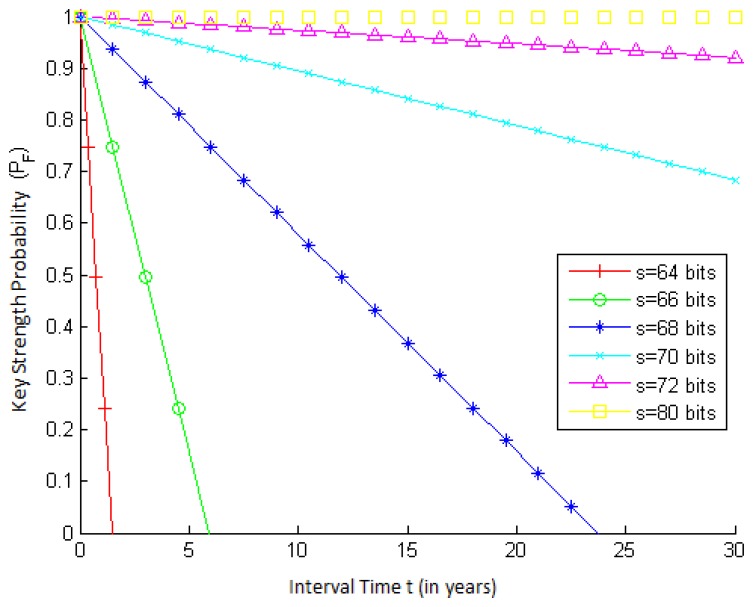
Key strength probability (*P_F_*) *vs*. time *t*, for different values of key strength *s* and rate *f* = 394, 875, 793, 722 keys/s.

Figure 2 presents how *P_F_* varies with time *t* for different values of key strength *s*. Notice that the greater the strength of a key, the more secure this key is, since its key space will be larger. On the other hand, *P_F_* decreases as time goes on, since more keys are tested, which increases the chances of an adversary breaking the algorithm. Furthermore, note that when t increases, *P_F_* decreases more steeply for smaller values of s. This is because for keys with smaller strengths, a greater fraction of the total key space is tested for a given value of *t*.

#### 4.3.2. Accuracy Analysis of *P_F_*

As *P_F_* represents the probability that a secret key has not been revealed by an adversary who performs a brute force attack, we compute the accuracy by comparing the estimate given by Equation (6) with the actual fraction (obtained from the simulations) of keys not discovered by an adversary yet. This fraction is measured in each simulation as follows. Each sensor node is equipped with a key of strength *s* randomly selected from the total space of 2^s^ keys. Then, a brute-force attack is performed in order to reveal the keys of the nodes, and at each time *t*, the fraction of nodes whose keys were not revealed yet is computed.

**Figure 3.**
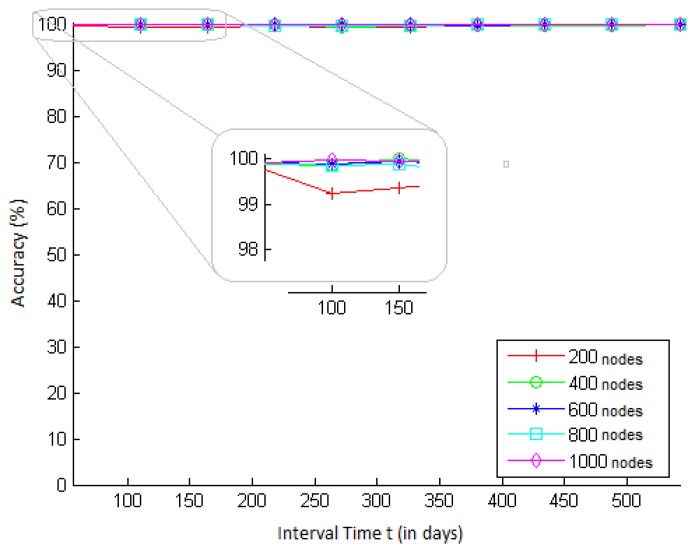
Accuracy of the key strength probability (*P_F_*) *vs*. time *t*, for different network sizes, for a test rate *f* = 394, 875, 793, 722 keys/s, and *s* = 64 bits.

We simulate scenarios with different network sizes and different values of *t*, for a key strength of *s* = 64 bits. The results obtained are presented in Figure 3. In this figure, we can see that the accuracy remained very high, between 99% and 100%, for all values of t and network sizes.

### 4.4. Resilience Probability (*P_R_*)

In this section, we first present the behavior of the metric *P_R_* according to Equation (7). Then, we present the accuracy achieved by *P_R_* in the simulations.

#### 4.4.1. Behavior Analysis of *P_R_*

To show the behavior of *P_R_*(*x*), the key management resilience probability as a function of the amount x of captured nodes, we use the q-composite scheme [22], because it is a widely-known key management mechanism, on which several others are based.

**Figure 4.**
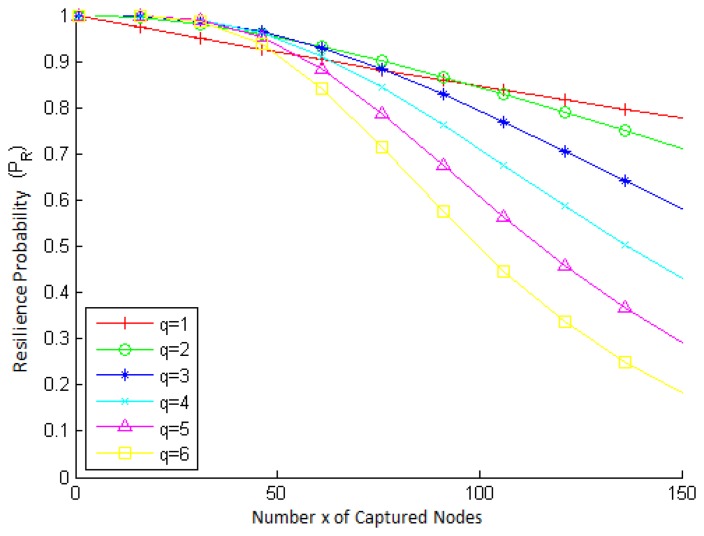
Key management resilience probability (*P_R_*) vs. amount x of captured nodes, for different values of key overlap *q*, *k* = 200 keys and *p* = 0.33.

Figure 4 presents how *P_R_* varies with *x*, for different values of *q* (the minimum amount of common keys necessary for establishing a secure link between any two nodes). Notice that the number of keys stored by each node is *k* = 200, and the connection probability between a node and its neighbors is *p* = 0.33. The key pool size |*K*| is computed from the values of *p*, *k* and *q*, according to Equations (9) and (10).

In Figure 4, we can see that up to about 50 captured nodes, the resilience probability remains close to one, for all values of *q*. However, for values of *x* greater than 50, *P_R_* decreases more significantly, besides being smaller for greater values of *q*. This is because when *q* is small, the nodes will share a smaller amount of common keys, which makes a node capture reveal little information about the other keys in the network. On the other hand, as *q* increases, a node capture has a far greater impact on the security of the network, causing more keys to be revealed. When the number of captured nodes is large, the negative impact on the security of the network is even more evident. Thus, it is possible to conclude that the q-composite scheme is not able to maintain the network as very secure when the number of captured nodes is very large.

#### 4.4.2. Accuracy Analysis of *P_R_*

As *P_R_* represents the probability that a secure communication link is not compromised after the capture of network nodes, we compute the accuracy by comparing the estimate given by Equation (7) (reminding that for the q-composite scheme, the *P_C_*(*x*) value used by Equation (7) is computed through Equation (11)) to the actual fraction (obtained from the simulation) of secure links that were not compromised after the capture of network nodes. This fraction is measured in each simulation as follows. Each sensor node is equipped with *k* keys out of the key pool *K* computed according to the respective values of *q* and *p*. Then, as soon as the secure links between the nodes are established, several nodes are captured one by one, and at each capture, the fraction of non-compromised links is computed.

**Figure 5.**
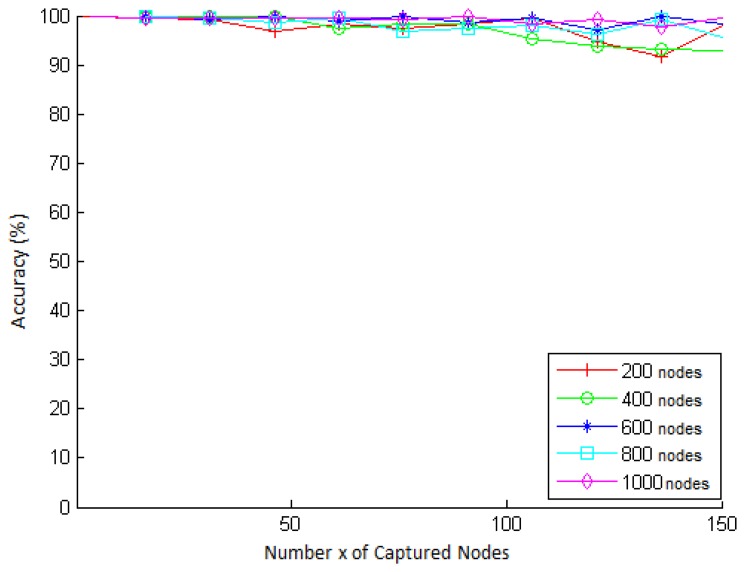
Accuracy of the key management resilience probability (*P_R_*) *vs*. the number *x* of captured nodes, for different network sizes, *k* = 200 keys, *p* = 0.33 and *q* = 3.

We simulate scenarios with different network sizes and different values of *x*, for *k* = 200, *q* = 3 and *p* = 0.33. The results obtained are shown in Figure 5. In this figure, we can see that the accuracy begins to vary more significantly from approximately 50 captured nodes, a situation in which the impact of node captures on the network links is larger, as shown in Figure 4. This is because the connection probability (*p* = 0.33) is set to the entire network. This means that it is expected that the network is 33% connected. However, since the connection model is probabilistic, each node may have an individual connection probability different from 0.33. Therefore, as the capture of a larger number of nodes has more impact on the existing links between other nodes, the more the fraction of connections of each neighbor deviates from the probability *p* = 0.33, the less is the accuracy when *x* is large. Nevertheless, the accuracy is still very high (greater than 90%) for all network sizes and values of *x*.

### 4.5. Legitimacy Probability (*P_L_*)

In this section, we evaluate the metric *P_L_* for the case that an alarm is raised against a node, that is *P_L_*(*alarm*). We first present the behavior of *P_L_*(*alarm*) according to Equation (19) (and Equations (13) and (14)). Then, we present the accuracy achieved by *P_L_*(*alarm*) in the simulations.

#### 4.5.1. Behavior Analysis of *P_L_*(*alarm*)

**Figure 6.**
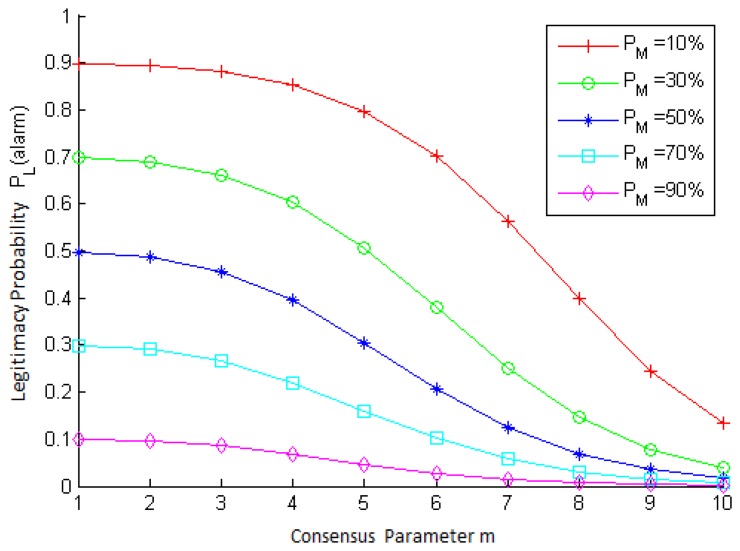
Legitimacy probability of a node when the intrusion detection system (IDS) generates a global alarm, *P_L_*(*alarm*), *vs*. the consensus parameter *m*, for different values of *P_M_*, *N* = 10, *P_tp_* = 0.6 and *P_fp_* = 0.4.

The behavior of the legitimacy probability equation, *P_L_*(*alarm*), is shown in Figure 6, for different values of *m* and *P_M_*, for *N* = 10 neighbors, *P_tp_* = 0.6 and *P_fp_* = 0.4. As we can see in Figure 6, *P _L_*(*alarm*) decreases when m increases, that is the more neighbors that are required for the IDS to decide that a node is malicious, the smaller the chance of a false alarm occurring. In other words, if a great amount of nodes raised an alarm against a node, then this nodes is more likely to be actually malicious. Notice, as well, that the chance of a false alarm occurring also decreases as the fraction of malicious nodes in the network (*P_M_*) increases.

#### 4.5.2. Accuracy Analysis of *P_L_*(*alarm*)

To validate the proposed metric *P_L_*(*alarm*), we simulate a network with 1000 nodes, each one of them with *P_tp_* = 0.6, *P_fp_* = 0.4 and *N* = 10 neighbors. We use different values for the consensus parameter *m* (from one to 10 neighbors) and for the base rate *P_M_* (10%, 30%, 50%, 70% and 90%), which results in a total of 50 scenarios. In this simulation, several nodes are randomly chosen to be malicious and perform attacks against the network. In addition, each node of the network is analyzed by its neighbors, which check, according to their individual hit and miss rates, whether they find that a node is malicious or not. In each scenario, 10, 000 IDS global evaluation processes (global decision-making processes of the IDS based on the individual outcome of each neighbor) are performed. At the end of each simulation scenario, *P_L_*(*alarm*) is computed by SDSE (according to Equation (19)) and compared to the actual fraction of legitimate nodes for which alarms were improperly generated.

**Figure 7.**
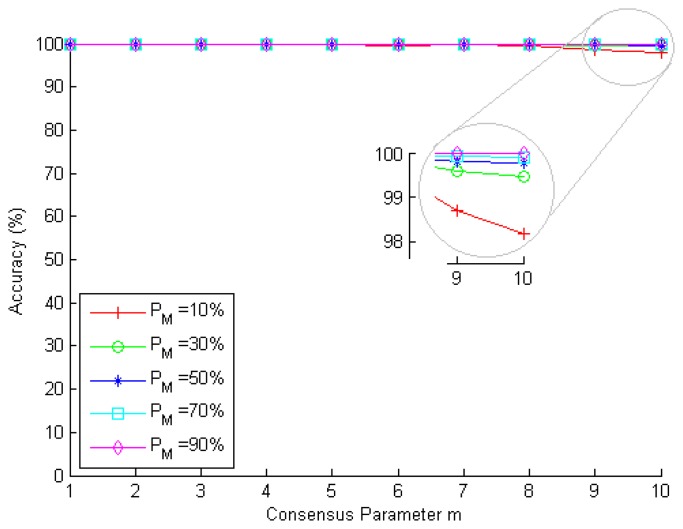
Accuracy of the legitimacy probability when the IDS raises a global alarm, *P_L_*(*alarm*), *vs*. consensus parameter *m*. For different values of *P_M_*, *N* = 10 neighbors, *P_tp_* = 0.6 and *P_fp_* = 0.4.

Figure 7 shows the accuracy of metric *P_L_*(*alarm*) obtained from the simulations performed. Notice that for all values of *P_M_* and for all values of m, the accuracy remained very high, between 98% and 100%. This is due to the assumption that the individual miss and hit rates used are the same for all nodes of the network. This is, indeed, a reasonable assumption, given that nodes usually use the same rules database (either attack signatures or normal network behavior profiles) to perform attack detection (although they do not necessarily achieve the same results at the same time due to the different view of the network that each node has). At last, we highlight that, since the metric *P_L_*(*alarm*) also depends on *P_M_*, the more accurate the value of *P_M_* (provided by offline analyses given by the IDS), the greater the accuracy of this metric.

### 4.6. Security Degree

In this section, we first present the behavior of the security degree *g*, according to Equation (21). Then, we present the accuracy achieved by *g* in the simulations.

#### 4.6.1. Behavior Analysis of *g*

To show the behavior of the security degree of a node, which results from the product of the security metrics computed by SDSE, we use the following configuration parameter values:

1. Cryptography: RC5 Algorithm.
  - *f* = 394, 254, 429, 396 keys/s;
  - *t* = 45 days;
  - *s* = 64 bits.
2. Key Management: q-composite scheme.
  - *k* = 200 keys;
  - *q* = 2 keys;
  - *p* = 0.33.
3. IDS: for the case when a global alarm is not raised by the IDS (*A*−).
  - *m* = 5 nodes;
  - *N* = 10 nodes;
  - *P_tn_* = 0.7;
  - *P_fn_* = 0.3.

**Figure 8.**
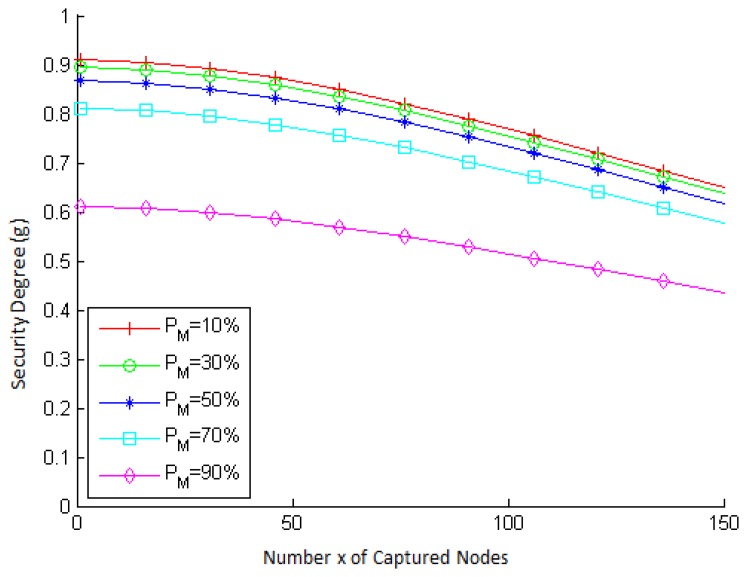
Security degree of a node for which a global alarm is not generated (*A*−) *vs.* the amount x of captured nodes, for different values of base rate (*P_M_*) and a network with 1000 sensor nodes.

Figure 8 presents the behavior of the security degree of a node as a function of the number x of captured nodes, for different values of base rate (*P_M_*) in a network with 1000 nodes. Note that the security degree decreases as *x* increases and that it decreases more significantly for values of *x* greater than 50. This behavior is expected, since it is the same behavior presented by the resilience probability *P_R_*(*x*). Notice, also, that the security degree is smaller for larger values of *P_M_*. Similarly, this behavior is inherited from the metric *P_L_*(¬*alarm*), in which the greater the number of malicious nodes in the network, the smaller the probability that a node is legitimate when an alarm is not raised against it.

#### 4.6.2. Accuracy Analysis of *g*

To validate the security degree estimated by SDSE, we simulate scenarios for different values of *x* (the number of captured nodes) and *P_M_* (the network base rate). Then, to compute the accuracy, we compare the security degree computed by SDSE (the product of estimated security metrics) to the actual security degree found in the simulations (the product of the fractions, regarding each metric, measured from the network).

**Figure 9.**
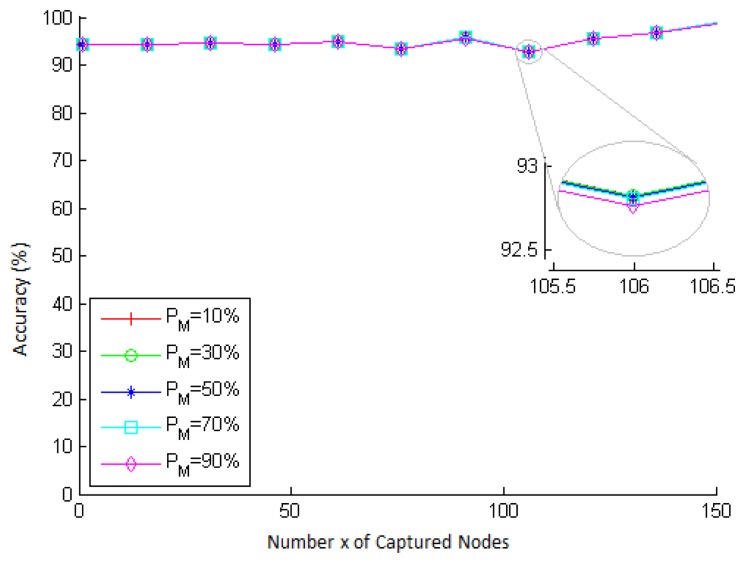
Accuracy of the security degree of a node for which a global alarm has not been generated (*A*−) vs. the number *x* of captured nodes, for different values of base rate (*P_M_*) and a network with 1000 nodes.

Figure 9 presents the accuracy obtained in simulations for 1000 nodes. In that figure, we can see that the accuracy is always greater than 92.5%. Notice that, as occurred with *P_R_*, the accuracy of the security degree has varied a little more significantly.

### 4.7. Security Level

As the main objective of SDSE is to assess the security of sensor data, simulations have been performed to validate, through accuracy, the security level (Equation (22)) estimated by SDSE for each query response message (carrying sensor data) delivered to the base station.

In each simulation, the accuracy of the security level estimated by SDSE is computed as follows. First, several data queries are disseminated throughout the network, from the base station. Then, all network nodes, upon receiving a query, send a respective response message. Each response message received at the base station, according to the route taken, has its respective security level computed by SDSE based on the security metrics proposed for each security mechanism. These messages are then grouped according to their security level. Notice that each message received at the base station may be either secure or corrupted. A secure message is a message that has not suffered any attacks during its way to the base station, *i.e.*, a message that was not: (1) forwarded by any node with the security key revealed by a brute force attack; (2) sent through any compromised link; or (3) forwarded by any malicious node (*M*+). On the other hand, a corrupted message is a message that has suffered any kind of attack on its way to the base station.

At the end of each simulation, for each possible security level (between zero and one), the fraction of secure messages is computed and compared with the respective estimated security level, so that we can calculate the accuracy. For example, suppose that during a simulation, 3000 response messages were delivered to the base station. Suppose also that, for 1000 of these messages, SDSE estimated a *SL* = 0.9. This means that SDSE expects that each of them is 90% likely to be secure. In other words, SDSE expects that, among these 1000 messages, 900 are secure and 100 are corrupt. Suppose also that, among the 2000 remaining messages, 1500 messages received a *SL* = 0.85 from SDSE and that 500 received a *SL* = 0.7, totaling 3000 messages delivered. Following that, out of the group of 1000 messages with *SL* = 0.9, the real fraction of secure messages was computed and compared to the respective security level estimated. That is, suppose among these 1000 messages, 910 are secure and 90 are corrupt. Therefore, the actual fraction of secure messages is 0.91, while SDSE estimated a fraction of 0.9, which results in an accuracy of $1-\frac{\left|0.91-0.9\right|}{0.91}\approx 98.9\%$ . Then, this same procedure for the calculation of the accuracy is performed for the 1500 messages with *SL* = 0.85 and for the 500 messages with *SL* = 0.7. At the end of the simulation, the accuracy considered for analysis was obtained by the average of the accuracies of each security level estimated for the messages.

The configurations used in the simulations to validate the SL estimation are the following:

1. Cryptography: RC5 algorithm.
  - *f* = 394, 254, 429, 396 keys/s;
  - *t* variable, from one to 542 days, randomly selected for each node;
  - *s* = 64 bits.
2. Key management: q-composite scheme
  - *k* = 200 keys;
  - *q* = 2 keys;
  - *p* = 0.33;
  - *x* = *5%* of the network nodes (randomly captured).
3. Intrusion detection system
  - *N*, variable for each node, depending on the network topology;
  - $m=\frac{N}{2}+1$ nodes;
  - *P_M_* = 10% of the network nodes;
  - *P_tp_* = 0.7;
  - *P_fp_* = 0.3;
  - *P_tn_* = 0.7;
  - *Pf_n_* = 0.3.

**Figure 10.**
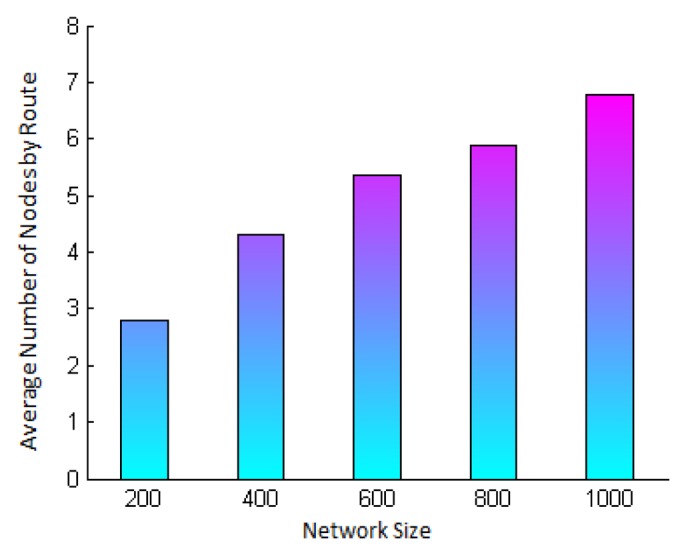
Average number hops to the base station, for different network sizes.

Before showing the accuracy results, we first show the average number of hops traveled by the messages in each simulated network (Figure 10), since the security level estimated takes into account the route traveled by the messages. See in this figure that, when the network size increases, the average length of the routes to the base station also increases. This figure allows us to see that the accuracy of the security level has been tested in networks with a minimum average route length of three hops and a maximum of seven hops.

**Figure 11.**
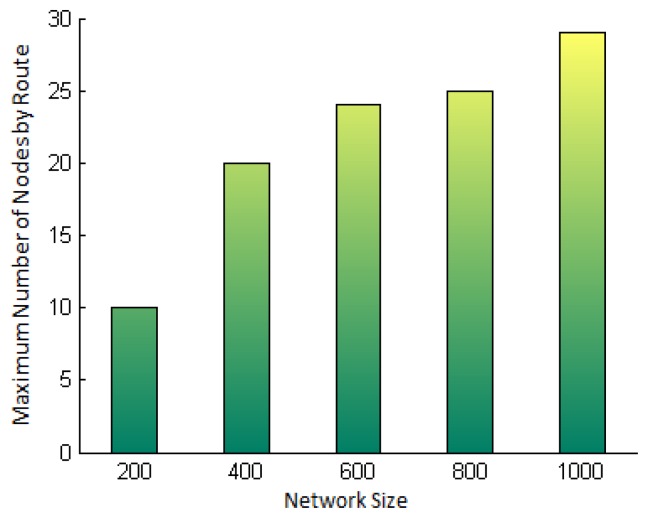
Maximum number of hops to the base station, for different network sizes.

In addition, Figure 11 presents the maximum number of hops (one hop being the minimum) traveled by the response messages in each simulated network. Notice that, in the network with 200 nodes, the farthest nodes are 10 hops away from the base station, and that in the network with 1000 nodes, the farthest nodes are up to 29 hops away from the base station.

**Figure 12.**
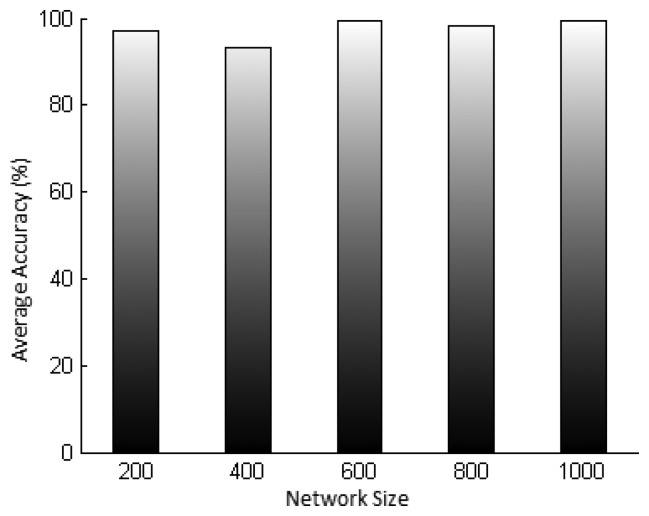
Accuracy of the estimated security level, for differently sized networks.

Finally, Figure 12 presents the average accuracy of SDSE estimates in differently sized networks. We can see that the accuracy remains extremely high for all network sizes, always next to 100%. Therefore, we conclude that the security level estimated by SDSE presents high accuracy for both messages that travel only one hop to the base station and messages that travel up to about 30 hops. This shows that the security level provided by SDSE is valid for both small and large networks.

### 4.8. Comparison of SDSE to Other Proposals

In terms of accuracy, we do not compare our proposed SDSE scheme with the mentioned schemes proposed for WSNs [5–8], because such schemes do not validate the metrics that they propose. However, in order to give a notion of how high the accuracies achieved by SDSE are, we compare our scheme with two of the main security level estimation schemes proposed for traditional networks [1,2], since they do validate their proposed metrics.

The work of Ahmed *et al.* [1] presents metrics with accuracies between 78% and 83%, while the scheme proposed by Li *et al.* [2] presents accuracies that vary between 55% and 100%. Regarding SDSE, as we have seen in the above subsections, its accuracies remained between about 90% and 100%.

From these values, we can see that SDSE presents accuracies similar to (or even better than) those of traditional networks' proposals.

## 5. Related Work

The systematic definition of security metrics and security level estimation are fields that are relatively new. To date, very few security estimators have been proposed for both traditional networks (e.g., [1–3]) and wireless sensor networks (e.g., [5–8]).

In traditional networks, to quantify security, most of the proposals are based on vulnerability and risk analysis (e.g., [1,44]) or, also, on the attack graph technique, used to model the relationship between multiple vulnerabilities [3]. These proposals use automated tools to identify vulnerabilities in the network and to quantify their severity based on the Common Vulnerability Scoring System [4]. Li *et al.* [2] present a stochastic model to quantify the security level that, in addition to using CVSS and attack graph, takes into account both the effectiveness and vulnerabilities of the security mechanisms deployed in the network.

For sensor networks, Hwang *et al.* [5] define the metric, security leakage factor, for quantifying security in WSNs based on a key management mechanism proposed by the authors themselves, called cluster key grouping. On the basis of the proposed mechanism, the authors define a value called the compromise factor, given by the number of keys compromised if a single node is compromised divided by the key pool size. Finally, they compute the security leakage factor by multiplying the compromise factor by a subjective value called the security weight, which can be any natural number that represents the importance that a security compromise has for the network.

Clark *et al.* [6] evaluate the security of a sensor network based on a metric for assessing vulnerability to key exposure of the key management mechanism, the link key security metric (LKSM). This metric allows one to analyze and compare the resilience of the key management and is defined as the time required for all of the keys comprising a secure connection to be captured, *i.e.*, the time required to fully compromise a connection. This value is obtained by uniformly capturing nodes at random, adding their cryptographic keys to a list of captured keys and then repeating this process, until all of the keys securing a given connection between two nodes have been captured. This metric is able to quantify the security of keys used for each connection, because if one key is used by a relatively small number of nodes (and is therefore more secure), a larger amount of nodes, on average, need to be captured until a node holding this key is captured.

Ksiezopolski and Kotulski [7] present a security model that uses several WSN protocol parameters to estimate security level. In the case that this level is below a certain threshold, the system security is dynamically optimized. In order to accomplish this, the authors assume a sensor network with high bandwidth connections, nodes that can detect incidents wherever in the network, and they propose equations that depend on several intuitively interpreted factors.

Sharma *et al.* [8] describe a WSN security framework comprised of key management, trust scheme, among others, and propose the intelligent security agent (ISA), a component to evaluate the security level based on node information, such as available memory, available energy, trust level and predefined policies, as well as recommendation. However, the authors do not provide any further details about how the proposed component uses this information to compute the security level.

## 6. Conclusions

With the increasing use of sensor networks and the large number of important decisions being made from sensed data, the need for a suitable scheme for estimating the data security level is more and more evident. In this context, the sensor data security estimator proposed may provide a significant contribution regarding security quantification in WSNs for being a scheme that takes into account both prevention and detection mechanisms and for defining metrics based on mathematical equations that use configuration parameters of the network itself and its mechanisms. This eliminates the need for assigning subjective weights, which are used in other existing proposals for WSNs. Furthermore, SDSE is able to evaluate the security of different types of cryptography algorithms, key management schemes and intrusion detection systems, differently from some of the other works, which are only focused on a single specific security mechanism.

Another aspect that makes SDSE stand out from other works is the fact that it has been validated through extensive simulations, presenting accuracies always between 90% and 100% (inclusive), for all of its estimates (security metrics, degree and level). Given the absence of validation for the existing security level estimation works proposed for WSNs, we have compared SDSE to security level estimation schemes proposed for traditional networks, such as the scheme proposed by Ahmed *et al.* [1], with accuracies between 78% and 83%, and the work proposed by Li *et al.* [2], with accuracies between 55% and 100%. From that comparison, we can conclude that SDSE is a highly accurate scheme, reaching accuracies similar to (or even better than) those of traditional networks' schemes.

Furthermore, as presented in Section 4, SDSE has been tested in a large amount of different scenarios, which allows us to conclude that SDSE is a viable scheme, can be used in both small and large networks and does not consume the energy of the sensors, being executed at the base station.

Finally, we emphasize that another major contribution of this work is the proposed security metrics and their equations, which, in addition to being used in real time by SDSE to estimate the sensor data security level, can also be used separately, in offline mode, to compare security mechanisms and help network administrators decide which mechanisms of cryptography, key management and intrusion detection will be installed on a network. SDSE metrics can also be used to tune the configuration of a given security mechanism and see its impact on the security of the network, as shown in the metrics behavior analysis presented in Section 4.
